# Defining Goal-Directed Training for Children with Cerebral Palsy: A Scoping Review and Framework for Implementation

**DOI:** 10.3390/children12081039

**Published:** 2025-08-08

**Authors:** Angela Shierk, Bridget Barry Thias, Haley Becker, Baylee Allen, Benjamin Chaiprasert, Katherine C. Lampe, Ava Wallace-McCollom, Aidan O’Brien, Heather Roberts

**Affiliations:** 1 Department of Clinical Research, Scottish Rite for Children, Dallas, TX 75219, USAbchaipra@student.touro.edu (B.C.); hroberts3@twu.edu (H.R.); 2 Department of Applied Clinical Research, UT Southwestern Medical Center, University of Texas, Dallas, TX 75390, USA; 3 Department of Occupational Therapy, Texas Woman’s University, Denton, TX 76204, USA

**Keywords:** cerebral palsy, goal-directed training, therapeutic intervention, outcome measures

## Abstract

**Highlights:**

**What are the main findings?**

- Defines and synthesizes the core components of Goal-Directed Therapy (GDT) for children with cerebral palsy through a comprehensive scoping review.
- Identifies measurable benefits of GDT across ICF domains, including motor function, self-care, communication, and participation, based on evidence from 112 intervention studies.

**What is the implication of the main finding?**

- Proposes a structured eight-step GDT framework to support therapists in implementing GDT with fidelity across settings and severity levels.

**Abstract:**

**Background/Objectives**: This scoping review aimed to define goal-directed training (GDT) and its impact on outcomes for children with cerebral palsy (CP), and to develop a structured framework outlining its core components for effective implementation. **Methods**: Using the Arksey and O’Malley framework and PICO criteria, nine databases were searched and reference lists reviewed. Two reviewers independently screened and extracted data, which were analyzed using a qualitative descriptive approach. **Results**: From 1273 articles, 156 met inclusion criteria, including 112 efficacy studies (53 randomized trials, 53 non-randomized trials, 6 secondary analyses) involving 4708 children aged 3 months to 21 years (mean age 6.7 years). Interventions addressed all GMFCS and MACS levels. Ninety outcome measures across ICF domains were used. GDT was associated with improvements in motor function, hand use, self-care, communication, and participation. Findings were synthesized into an eight-step GDT framework highlighting collaborative goal setting, goal analysis, strategy determination, structured practice, feedback, re-evaluation, and generalization. This framework supports consistent, high-quality GDT implementation across settings and disciplines. **Conclusions**: In conclusion, GDT shows broad functional benefits and emphasizes individualized, client-centered care. The review offers a practical, evidence-informed framework to guide clinicians and researchers in delivering GDT with fidelity.

## 1. Introduction

Cerebral palsy (CP) is a group of permanent disorders that affect movement and posture, caused by nonprogressive disturbances to a child’s brain during pregnancy or infancy [1]. Individuals with CP may also experience epilepsy, secondary musculoskeletal problems, and impairments in sensation, perception, cognition, and behavior, all of which can limit daily activities [1]. Current literature highlights goal-directed training (GDT) as a method to improve function in children with CP, where the child actively practices their desired goal or task [2].

Training-based interventions such as GDT, which use varied and repetitive activities focused on a child’s specific goals to promote neuroplasticity and improved function, are considered standard for improving function in children with CP [3]. While GDT is effective, there remains a gap between evidence and clinical implementation, highlighting the need for better knowledge translation strategies for healthcare professionals [4]. Current literature shows variability in how GDT is described and delivered, with limited guidance on its core components [5,6]. Without clear protocols or frameworks, providers struggle to implement GDT as intended [5]. This article addresses this gap by defining GDT and evaluating its impact on measurable outcomes for children with CP through a scoping review, ultimately proposing a framework that outlines its core components to support consistent and effective practice.

## 2. Materials and Methods

### 2.1. Protocol and Registration

To systematically identify and review the literature, we utilized the five-step scoping review methodological framework developed by Arksey and O’Malley [7]. This scoping review was conducted in accordance with the PRISMA-ScR (Preferred Reporting Items for Systematic reviews and Meta-Analyses extension for Scoping Reviews) checklist to ensure methodological rigor and transparency. The protocol for this review was registered on the Open Science Framework [8].

### 2.2. Eligibility Criteria

Inclusion criteria included peer-reviewed articles from the past 20 years that describe goal-directed or goal-oriented training or therapy as an intervention for youth with CP. Youth includes infants, toddlers, preschoolers, children, and adolescents. Studies not in English or lacking full-text access were excluded. Studies not published in English were excluded due to the language proficiency of the review team, which may introduce language bias. While translation tools were considered, the team determined that relying on automated or third-party translations could compromise accuracy and interpretation, especially for nuanced clinical or contextual findings. as the reviewers are English-speaking only and the timeframe of 20 years was adequate to establish the content needed.

### 2.3. Information Sources

Authors were not contacted; however, reference lists of systematic reviews were reviewed to locate individual articles. The first search was in September 2023 with the last in December 2023. Databases searched included PubMed, Academic Search Complete (EBSCO), CINAHL Complete (EBSCO), Nursing and Allied Health (Proquest), Cochrane, Scopus, Psychology Database (ProQuest), Google Scholar, and Up to Date.

### 2.4. Search Strategy

Search terms were selected based on an initial search for GDT and expanded to include related terms commonly used in the literature. Consistent search strands were used across databases for simplicity and consistency. Terms included “goal-directed therapy” OR “goal-oriented therapy” OR “goal-oriented training” OR “goal-directed training” and “cerebral palsy”. The MESH term for “cerebral palsy” was used for PubMed, Academic Search Complete and CINAHL Complete. Additional articles were identified by manually reviewing the reference lists of articles that met inclusion criteria and searching the first author’s name using the author tab in Scopus. The search strategy was validated by confirming that articles citied in two key systematic reviews identifying GDT as an evidence-based intervention for children with [9,10] were captured. Each database was searched individually, and the number of articles retrieved from each was documented.

### 2.5. Selection of Sources of Evidence

All articles retrieved were imported into the reference management system Covidence, where duplicates were removed. Two independent reviewers completed each stage of the review process, with a third reviewer verifying the results. The review stages included (1) review of titles and abstracts, (2) full text review, and (3) data extraction. Prior to initiating the review, inclusion and exclusion criteria were discussed among the team. To enhance consistency and reduce bias, reviewers engaged in calibration exercises using a subset of articles and met regularly to discuss the application of criteria. All screening and extraction were conducted in real time by two reviewers, with consensus reached collaboratively and adjudicated by a third reviewer when needed. Team meetings occurred biweekly throughout 2024 to discuss article eligibility, ensure reliability, and maintain rigor in the screening and data extraction process. Articles that met the inclusion criteria were analyzed to identify and summarize the key components of GDT and its impact across domains of the International Classification of Functioning, Disability and Health (ICF).

### 2.6. Data Charting Process

The following information was extracted into Covidence from each article: first author, date of publication, short title, population, intervention, comparator, perception of intervention, outcome measures, results, key findings, and additional comments. Population data extracted included diagnoses, age (range and mean), gender, Gross Motor Function Classification System (GMFCS) levels, Manual Ability Classification System (MACS) levels, and other classification data. It was also noted whether multiple diagnoses were included and if results were reported separately or combined. Descriptions of the intervention included dose and key components. Perceptions from participants, caregivers, and therapists were included when available. Outcome measures were identified along with the corresponding ICF domain assessed. For quantitative studies, the impact of the intervention on each outcome measure was assessed, focusing specifically on areas where GDT demonstrated a positive effect. For qualitative studies, key findings and themes were summarized. From January to April 2025, the research team continued to meet biweekly for data analysis and synthesis, engaging in frequent discussions to ensure consistent and thoughtful coding. This iterative process supported the development and validation of themes through consensus. As a final validity check, reviewers confirmed whether each article contained relevant information on GDT or its impact.

### 2.7. Synthesis of Results

Extracted data were summarized and categorized including demographic information, frequency of outcome measures, and improvements. Outcome measures showing improvement were listed and totaled to describe the overall impact of the intervention. Using qualitative descriptive approach [11], two reviewers identified seven key themes of GDT based on intervention descriptions across articles. These themes were validated by counting how frequently they appeared across the included articles. The identified GDT themes, combined with clinical expertise, informed the development of a step-by-step therapeutic process to guide healthcare professionals in implementing GDT.

## 3. Results

### 3.1. Selection of Sources of Evidence

**Figure 1:**
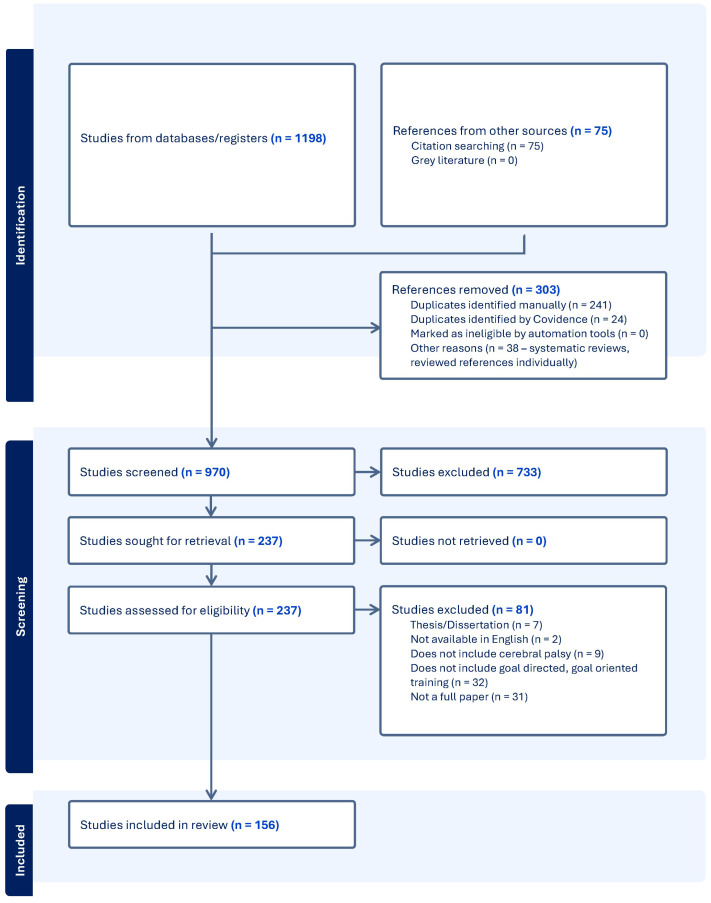
PRISMA flow diagram.

The initial database search yielded 1198 articles, with an additional 75 articles identified through citation searching for a total of 1273 articles. After removing duplicates, 970 studies remained for screening. Following the title and abstract review, 237 full-text articles were assessed for eligibility. Of these, 81 were excluded, and a total of 156 articles met inclusion criteria (Figure 1).

### 3.2. Characteristics of Sources of Evidence

Of the 156 studies, 112 reported on the efficacy or effectiveness of interventions including 53 randomized trials, 53 non-randomized trials, and 6 post hoc or secondary analyses.

### 3.3. Study Participants

The 112 quantitative studies included 4708 participants from 3 months to 18 years old with a mean age of 6 years and 8 months (SD 1 year and 3 months). Participants included 2560 males and 1738 females. There was representation of participants at each GMFCS and MACS level, although most participants fell within levels I-III (Table 1).

### 3.4. Outcome Measures and ICF Domains

**Figure 2:**
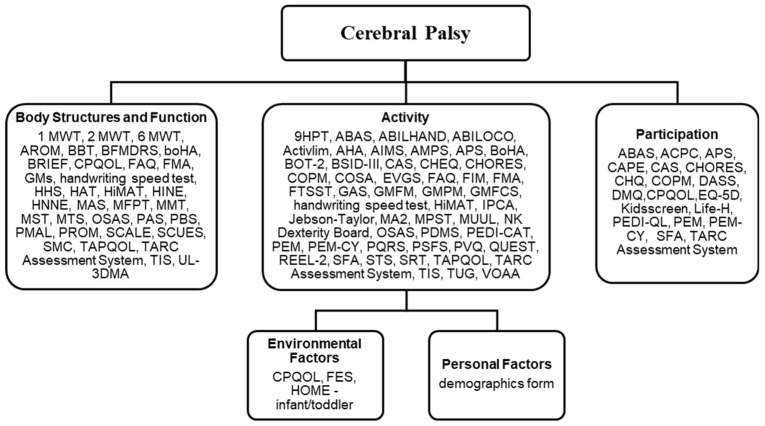
Outcome measures by ICF domain. **Abbreviations:** 1-MWT, One Minute Walk Test; 2-MWT, Two Minute Walk Test; 6-MWT, Six Minute Walk Test; 9HPT, Nine Hole Peg Test; ABAS, Adaptive Behavior Assessment System; ABILHAND, Manual Ability Measure for Children with Upper Limb Impairments; ABILOCO, Abilities in Locomotion Questionnaire; ACPC, Assessment of Preschool Children’s Participation; Activlim, Activity Limitations Questionnaire; AHA, Assisting Hand Assessment; AIMS, Alberta Infant Motor Scale; AMPS, Assessment of Motor and Process Skills; APS, Assistance to Participate Scale; AROM, Active Range of Motion; BBT, Box and Block Test; BFMDRS, Burke-Fahn-Marsden Dystonia Rating Scale; BoHA, Both Hands Assessment; BOT-2, Bruininks-Oseretsky Test of Motor Proficiency, Second Edition; BRIEF, Behavior Rating Inventory of Executive Function; BSID-III, Bayley Scales of Infant Development, Third Edition; CAPE, Children’s Assessment of Participation and Enjoyment; CAS, Caregiver Assistance Scale; CHEQ, Children’s Hand-use Experience Questionnaire; CHORES, Children Helping with Responsibilities, Expectations, and Supports; CHQ, Child Health Questionnaire; COPM, Canadian Occupational Performance Measure; COSA, Child Occupational Self-Assessment; CPQOL, Cerebral Palsy Quality of Life Questionnaire; DASS, Depression Anxiety Stress Scales; DMQ, Dimensions of Mastery Questionnaire; EQ-5D, EuroQol 5-Dimension Questionnaire; EVGS, Edinburgh Visual Gait Score; FAQ, Functional Assessment Questionnaire; FES, Family Environment Scale; FIM, Functional Independence Measure; FMA, Fugl-Meyer Assessment; FTSST, Five Times Sit to Stand Test; GAS, Goal Attainment Scaling; GMFCS, Gross Motor Function Classification System; GMFM, Gross Motor Function Measure; GMPM, Gross Motor Performance Measure; GMs, General Movements; Handwriting Speed Test, Handwriting Speed Test; HAT, Hypertonia Assessment Tool; HHS, Harris Hip Score; HiMAT, High-level Mobility Assessment Tool; HINE, Hammersmith Infant Neurological Examination; HNNE, Hammersmith Neonatal Neurological Examination; HOME—Infant/Toddler, Home Observation for Measurement of the Environment—Infant/Toddler Version; IPCA, Inventory of Ptential Communicative Acts; Jebson-Taylor, Jebson-Taylor Hand Function Test; Kidsscreen, KIDSCREEN Health-Related Quality of Life Questionnaire for Children and Adolescents; Life-H, Assessment of Life Habits; MA2, Melbourne Assessment 2; MAS, Modified Ashworth Scale; MFPT, Manual Form Perception Test; MMT, Manual Muscle Testing; MPST, Muscle Power Srint Test; MST, Meter Sprint Test; MTS, Modified Tardieu Scale; MUUL, Melbourne Assessment of Unilateral Upper Limb Function; OSAS, Observational Skills Assessment Score; PAS, Postural Assessment Scale; PBS, Pediatric Balance Scale; PDMS, Peabody Developmental Motor Scales; PEDI-CAT, Pediatric Evaluation of Disability Inventory—Computer Adaptive Test; PEDI-QL, Pediatric Quality of Life Inventory; PEM, Participation and Environment Measure; PEM-CY, Participation and Environment Measure for Children and Youth; PMAL, Pediatric Motor Activity Log; PQRS, Performance Quality Rating Scale; PROM, Passive Range of Motion; PSFS, Patient-Specific Functional Scale; PVQ, Pediatric Volitional Questionnaire; QUEST, Quality of Upper Extremity Skills Test; REEL-2, Receptive-Expressive Emergent Language Test, Second Edition; SCALE, Selective Control Assessment of the Lower Extremity; SCUES, Selective Control of the Upper Extremity Scale; SFA, School Function Assessment; SMC, Selective Motor Control; STS, Sit to Stand Test; SRT, Shuttle Run Test; TAPQOL, TNO-AZL Preschool Children Quality of Life Questionnaire; TARC Assessment System, Topeka Association for Retarded Citizens Assessment System; TIS, Trunk Impairment Scale; TUG, Timed Up and Go Test; UL-3DMA, Upper Limb 3-Dimensional Motion Analysis; VOAA, Video Observations Aarts and Aarts.

The ICF framework includes five components: body structures/functions, activity, participation, personal factors, and environmental factors [12]. While some outcome measures assessed more than one domain, the majority assessed activity, with fewer addressing body structures/function and participation (Figure 2).

Demographic data was the only personal factor measure identified. No outcome measures were used to exclusively measure environmental aspects, though some included supplemental questions on the environment. Ninety outcome measures were used across the 112 intervention studies. The Canadian Occupational Performance Measure (COPM), Goal Attainment Scale (GAS), Gross Motor Function Measure (GMFM), the Pediatric Evaluation of Disability Inventory (PDEI), and the Assisting Hand Assessment (AHA) were the most frequently used outcome measures (Table 2). For an overview of outcome measures and the ICF domains identified, refer to Supplemental Material Table S1.

### 3.5. Impact of GDT

GDT led to improvements in self-care [2] and motor outcomes including dexterity, grip, functional hand-use, balance, and walking endurance [2,19,47,63,66], and is recommended over other interventions to address underlying impairments [2]. Gross motor and quality of life outcomes were better in GDT groups than those in traditional therapy [68]. Children who participated in GDT, especially group-based settings, showed increased participation and motivation throughout intervention [54,63,71,115,116]. Mobility improved across GMFCS levels when using whole-task practice and real-life contexts [2]. GDT improved social performance by increasing opportunities for communication and problem-solving, encouraging children to be active participants in expressing with their feelings, solutions, and preferences [75]. Caregivers expressed that their child’s achievements exceeded their expectations, and they felt more knowledgeable and satisfied in their ability to support their child toward future goals [75,117].

### 3.6. Key Themes Identified in GDT Intervention

The key themes of GDT were identified and validated through a frequency count of their occurrence across articles (Table 3). These themes included: collaborative goal setting, family-centered practice, specific training techniques, therapy dose, social engagement, multidisciplinary approaches, and outcome monitoring.

**Theme** **1.** *Collaborative Goal Setting.*

Collaborative goal setting involves working with the child and family to establish intervention goals [16,44,118]. This approach, which includes family-centered and child-directed goal setting, aims to enhance functional performance and promote independence in daily activities [68,83]. Approximately 79 percent of the articles included collaborative goal setting (Table 3). Out of those 124 articles, 44 included the COPM and 41 the GAS (Table 2) as an aid in goal setting. Additionally,15 articles used interviewing prior to intervention to establish functional goals [48,75,76,77,78,79,81,82,83,86,90,91,100,108,153].

**Theme** **2.** *Family-Centered Practice.*

Family-centered practice prioritizes the family’s self-determination, decision-making capacity, and self-efficacy throughout the therapeutic process [14,37,44,70,120]. Key aspects of this approach include family-focused strategies, the family’s role in therapy, effective communication, and family education [120]. Approximately 58% of the articles reviewed discussed family-centered practice (Table 3).

**Theme** **3.** *Specific Training Techniques.*

Specific training techniques for GDT typically involve an individualized programs tailored to each child’s goals [2,16,18]. These techniques are often described in detail to guide intervention planning and implementation [156]. In this review, 67% of the included articles provided descriptions of specific training techniques (Table 3). Among these, constraint-induced movement therapy (CIMT) and bimanual therapy were the most frequently utilized.

CIMT involves constraining the less impaired or unaffected hand to encourage intensive practice with the affected hand during unilateral tasks [28,48,49,61,94,109]. CIMT involves repetition and shaping [99] and has well-established positive effects in children with unilateral CP [99]. Bimanual therapy, another common training technique utilized for children with CP, involves intense repetitive practice using both hands during bilateral tasks [28,99] to promote daily functioning [54]. HABIT focuses on intensive, progressive training [24,94] to improve bimanual coordination through functional activities and play [95,102] to increase independence [93]. These specific training techniques improve hand function in children with CP [102] and are among the most used approaches as they reduce limitations through daily practice in their natural environment [99].

**Theme** **4.** *Therapy Dose.*

Therapy dose refers to the length and frequency of intervention sessions [2,124,140,141,164]. Approximately 76% of the articles included a documented therapy dose or a comparison of doses (Table 3). Of those 119 articles, 89 were intervention studies with a documented dosage, while 31 were qualitative or descriptive studies that discussed dosage. Dosages ranged from approximately two hours over six months in a non-randomized experimental study [147] to the maximum dosage of 479 h over 3 years in a long-term repeated interval rehabilitation study [90]. The average therapeutic hours spent was 53.5 (median = 42). The average intervention duration was 9 weeks (median = 6 weeks).

**Theme** **5.** *Social Engagement.*

Social engagement encompasses interpersonal interactions that occur during activity [44,52,71,138]. Of the 156 articles included in the review, approximately 19% discussed social engagement or used primary or secondary outcome measures that assessed social engagement (Table 3). The social environment fostered engagement through approaches including group-based interventions, camp-based models, and community-integrated interventions. The social environment can also be reduced to support skill development, in these scenarios it is important to practice the skill across contexts, including social context to meet mastery.

**Theme** **6.** *Multidisciplinary Approaches.*

Multidisciplinary approach is the collaboration of healthcare professionals and other individuals involved in the child’s GDT [50,156] to target specific skills needed within their daily routine [31,161]. Within multidisciplinary approaches, home and school-based therapies were identified. Healthcare professionals are often the first team members individuals consider; however, caregivers are also a key component of this team for their ability to increase their child’s potential within a home-based program [131]. In school-based therapy settings, physical therapists, occupational therapists, and speech language pathologists [64] often collaborate with teachers and teaching assistants to create group interventions tailored to children’s goals, identified through outcome assessments [63]. Multidisciplinary approaches were included in 30% of the articles (Table 3). Out of those 47 articles, 31 included home-based therapy and 25 school-based as approaches to intervention using a multidisciplinary approach.

**Theme** **7.** *Outcome Monitoring.*

Outcome monitoring refers to tracking a child’s progress during the intervention, using feedback loops between caregivers and clinicians or assessment during the intervention to adjust the treatment as needed [2,40,66,77,131]. Approximately 15% of the articles incorporated outcome monitoring, either through ongoing progress assessments or structured feedback mechanisms during the intervention (Table 3). Fourteen of these studies included outcome measures during the intervention phase [2,14,37,39,40,41,43,63,66,67,69,75,77,78,99,108,113,114,118,131,136,147,152,160].

### 3.7. GDT Framework

**Figure 3:**
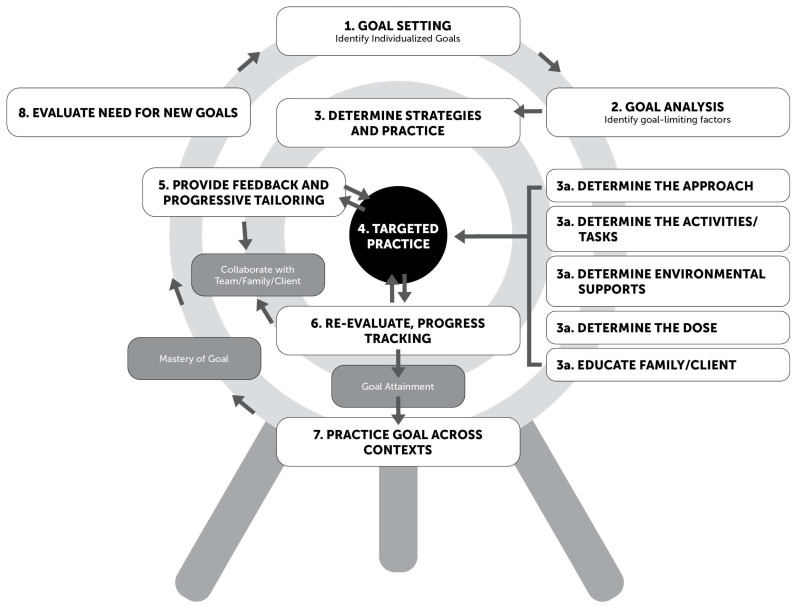
Goal-directed training framework.

Based on the identified and validated themes, a structured Goal-Directed Training (GDT) Framework was developed to guide healthcare professionals in delivering GDT with consistency and fidelity. The framework synthesizes the core components found across the literature and integrates clinical expertise to outline a stepwise therapeutic process. This framework is intended to support individualized, evidence-based intervention planning for children with cerebral palsy. The core components of the GDT Framework are defined in Table 4 and visually represented in Figure 3.

## 4. Discussion

While GDT is effective for children with CP [3], there is a need for knowledge translation and a clear guide for implementation strategies to ensure that healthcare providers are providing the most effective interventions available [4]. This article defines GDT and its impact on measurable outcomes for children with CP through a scoping review resulting in the development of a framework that more clearly describes its core components to help providers carry out GDT more effectively.

### 4.1. Collaborative Goal Setting

Collaborative goal setting engages caregivers in creating realistic, meaningful goals. As experts on their child’s needs, caregivers play a vital role in care decisions [83]. This collaborative approach ensures interventions align with the child’s needs while respecting the family’s values, preferences, and worldview. In addition to the caregiver perspective, child-directed goal setting focuses on the child’s interests and needs, aiding their engagement and motivation in the GDT process. When interventions focus on the child’s goals, they help build a sense of competency and self-efficacy [42], motivating the child to try new tasks, even those they previously found challenging.

The theme of collaborative goal-setting directly informed steps 1 and 8 of the GDT Framework. Step 1 focuses on identifying and setting individualized, function-based goals through a collaborative approach targeting meaningful activities. Step 8 emphasizes reassessing and setting new goals in collaboration with the child and family. The involvement of both caregiver and child perspectives is central to collaborative goal setting and reinforces a family-centered approach throughout the GDT process.

### 4.2. Family-Centered Practice

Promoting family-centered practice requires ongoing communication and empowerment throughout the intervention process. Feedback mechanisms, such as home diaries, home programs, and frequent caregiver contact, support collaborative problem-solving and help ensure skills transfer to the home environment [14]. These strategies also create opportunities for caregivers to share input on progress and guide necessary adjustments. Family-centered approaches should intentionally include efforts to build caregiver capacity and promote empowerment. Such interventions have been shown to reduce burden and stress [68], while also increasing caregivers’ confidence and understanding of their child’s potential [75].

Family-centered principles are embedded throughout the GDT Framework. Steps 1–3 emphasize the family’s role in setting meaningful goals and planning interventions. Steps 4–8 maintain the family’s position as a key team member through caregiver training, inclusion in outcome monitoring, and opportunities for feedback and program adjustments. Active family involvement is essential and is supported through consistent training, communication, and guidance.

### 4.3. Specific Training Techniques

Specific training techniques reduce a child’s limitations and support increased participation in their natural environment [99]. Before implementation, both the family and the child must receive thorough education and feel confident in the approach. For safety and effectiveness, these techniques should only begin once confidence is established. A key factor in the success of a specific training techniques is the collaboration between the family and the provider. Together, they identify meaningful, goal-directed activities [18] and adjust the intensity and duration of training as needed over time [16,99].

These principles inform steps 3–8 of the GDT Framework, where the child engages in targeted practice toward a specific skill or goal using techniques selected by a multidisciplinary team. In step 3, the provider works closely with the family and child to design a personalized plan that reflects with the child’s individual needs. The provider also fosters motivation and commitment by creating an optimally challenging practice environment, clearly defining the therapy dose, and ensuring the family understands how to carry out the techniques effectively. Importantly, therapy dosage plays a critical role in the overall success of specific training techniques.

### 4.4. Therapy Dose

Optimal therapy dosage depends on several factors, including the child’s individual needs, the complexity of the goal, type of intervention, available resources, and context of care [2]. Although the precise dose required to achieve long-term improvements in upper limb motor outcomes remains uncertain, previous systematic reviews suggest a target of 30–40 h over 6 months [140,141]. High-dose programs such as CIMT and bimanual training are well supported by evidence, while smaller-dose interventions have shown limited effects on upper-limb motor function is limited [2,124,141]. A full dose of CIMT or bimanual therapy is typically necessary for motor improvements, though a half dose may still support gains in occupational performance [47]. Notably, GDT has demonstrated positive effects on activity, participation, and motor function even at a lower therapeutic doses [52,123].

Despite these findings, dosage decisions are often shaped more by system-level constraints than by multidisciplinary team recommendations, frequently resulting in suboptimal dosing for neuroplastic change [131]. When aligned with family preferences and evidence-based practice, home programs can be an effective way to increase therapy dosage and overcome implementation barriers [127,131,164].

These insights informed several steps of the GDT Framework. In step 3d, providers determine the therapy dosage based on the family needs, environmental factors, and the outcome goals. In step 4, the established dose is implemented. In steps 5 and 6, dosage may be adjusted based on progress monitoring and feedback from the child, family, and team.

### 4.5. Social Engagement

Children with CP typically engage in leisure and recreation that is less varied, less social, and more sedentary compared to their peers [48,69]. While social engagement is a key factor influencing participation in school, activities, and leisure, it is often under-assessed or treated as a secondary outcome in research. Group-based interventions, however, offer a valuable strategy, supporting individual goal achievement within shared social activities. These settings naturally promote social interaction, motivation, and participation [14,63,86]. Group play and peer interaction help children develop social skills organically, while also enhancing activity levels and motor function [44,83]. Improvements in social engagement through GDT may reflect the child’s abilities to apply problem-solving skills in social contexts or result from increased physical activity stimulating social growth [65]. Because of this, social engagement should be an intentional part of intervention planning, especially in group settings, which offer a natural and indirect method for fostering these skills [75].

These considerations informed steps 2–7 of the GDT Framework. In steps 2 and 3, providers assess social factors as potential barriers or facilitators in goal achievement and identify environmental supports, including social contexts. In steps 4–7, GDT principles are applied to encourage skill acquisition, particularly when goals are social in nature or occur in social environments. Throughout this process, alignment with multidisciplinary team ensures that social engagement opportunities are integrated intentionally and effectively into treatment planning.

### 4.6. Multidisciplinary Approaches

Home-based therapy supported by a multidisciplinary team typically involves coaching caregivers to deliver daily interventions [145]. Each team member plays an active role in ensuring that the prescribed interventions are implemented effectively and aligned with the child’s goals [145]. Through the training and knowledge provided by the multidisciplinary team, parents gain the confidence and skills needed to support their child’s therapy at home [130].

In school-based therapy, cross-disciplinary collaboration involves professionals from different fields working together to support a child’s progress toward academic and school-related goals. Therapists offer ongoing advice and support to the teachers and staff through regular visits, guided by the GDT process [75]. By the end of the intervention, school-based professionals often report increased knowledge in motivating and training children using the GDT process [75].

These examples of multidisciplinary care informed the development of each step in the GDT Framework, highlighting the importance of continuous involvement and communication within the multidisciplinary to provide comprehensive care. A coordinated, multidisciplinary approach ensures that the child’s goals are addressed through individualized interventions. Regular outcome monitoring further supports the effective implementation of GDT.

### 4.7. Outcome Monitoring and Alignment with ICF Domains

Outcome monitoring emerged as a critical component of the GDT Framework, with two central subthemes: (1) using progress monitoring assessments during the intervention period and (2) engaging caregivers through feedback to review progress and adjust the intervention accordingly. Tracking outcome measures enables clinicians to monitor progress and enhance sensitivity to intervention efficacy [66]. Regular feedback allows caregivers to stay informed about their child’s progress and helps clinicians identify improvements, adjust difficulty, and tailor the program to stimulate continued progress [40,131]. Programs that enact strategies for ongoing reflection and performance reporting can foster a sense of competence, achievement, and motivation, supporting sustained engagement goal-directed activities [136]. Caregiver feedback can be delivered in various formats, including face-to-face updates, phone calls, video check-ins, or email [2].

Outcome monitoring is embedded throughout the GDT Framework. In step 2a, providers establish baseline performance using standardized outcome measures. In step 5, feedback between the therapy team and family informs real-time adjustments to the intervention. Step 6 continues this feedback loop following mid-program assessment, and in step 8, post-intervention assessment data is obtained and analyzed to determine whether new goals should be established.

Selection of outcome measures should be guided by a clear understanding of the intervention’s purpose, which can be effectively informed by the International Classification of Functioning, Disability and Health (ICF) framework [130]. The ICF provides a shared language for describing health and functioning across disciplines and has helped shift the focus of therapy from isolated impairments to a holistic, participation-centered approach [2]. While many interventions for children with CP target activity-level outcomes, a substantial number still focus on body structures and functions [22]. The GDT Framework encourages thoughtful selection of outcome measures aligned with meaningful goals across ICF domains, reinforcing the importance of personalized, goal-directed therapy.

### 4.8. Limitations

This scoping review includes some limitations. First, multiple reviewers were involved in the screening and data extraction stages, which may have introduced variability and subjectivity in the interpretation of findings. Second, the search was limited to articles published in English within the past 20 years, potentially excluding relevant studies outside this timeframe or in other languages. This decision reflects a balance between comprehensiveness and feasibility, and is a recognized limitation of the review. Future research may consider including non-English studies with appropriate multilingual expertise to expand global representation. Although the initial search was completed in 2023 and yielded a large number of articles, screening, extraction, and synthesis continued throughout 2024 and early 2025. Due to limited resources, the literature search was not updated beyond this period, and newly published studies may not have been captured. This is acknowledged as a limitation, and future updates of this review may consider including more recent publications to enhance currency. Additionally, gray literature was not included, as the volume of peer-reviewed results was sufficient for addressing the aims of the scoping review.

## 5. Conclusions

This scoping review identified seven key themes central to goal-directed therapy (GDT): collaborative goal setting, family-centered practice, specific training techniques, therapy dose, social engagement, multidisciplinary approaches, and outcome monitoring. These themes were consistently reflected across a wide range of studies and disciplines, forming the foundation for a comprehensive GDT Framework. This framework outlines both the step-by-step process and specific guidelines needed to help healthcare professionals implement GDT with fidelity.

Evidence suggests that GDT positively impacts multiple domains, including motor function, self-care, communication, problem-solving, parental knowledge and confidence, and child motivation. However, variability in how GDT is implemented has contributed to a gap between research and practice. By clearly defining the core components and therapeutic process, this review provides a practical, systematic approach for integrating GDT into routine care. The resulting framework offers clinicians and researchers a shared model to support intervention planning, delivery, and evaluation, ultimately enhancing functional outcomes for children with CP.

## Figures and Tables

**Table 1:** Demographics.

(*n* = 4708)	*n*	%
Gender		
Male	2560	54%
Female	1738	37%
Not reported	410	9%
GMFCS		
I	735	16%
II	597	13%
III	515	11%
IV	355	8%
V	190	4%
Not reported	2316	49%
MACS		
I	433	9%
II	916	19%
III	327	7%
IV	68	1%
V	63	1%
Not reported	2901	62%
	Mean	SD
Age	6.8 years	1.3 months

**Abbreviation:** GMFCS: Gross Motor Classification System; MACS: Manual Ability Classification System.

**Table 2:** Most commonly used outcome measures and source citations.

Outcome Measure	Count	Citations
Canadian Occupational Performance Measure (COPM)	44	[13,14,15,16,17,18,19,20,21,22,23,24,25,26,27,28,29,30,31,32,33,34,35,36,37,38,39,40,41,42,43,44,45,46,47,48,49,50,51,52,53,54,55,56]
Goal Attainment Scale (GAS)	39	[13,14,15,20,23,29,30,31,32,33,34,35,36,39,40,43,44,46,52,53,56,57,58,59,60,61,62,63,64,65,66,67,68,69,70,71,72,73,74]
Gross Motor Function Measure (GMFM)	37	[16,17,19,31,36,41,45,46,52,55,59,63,64,65,66,68,69,72,73,75,76,77,78,79,80,81,82,83,84,85,86,87,88,89,90,91,92]
Pediatric Evaluation of Disability Inventory (PEDI)	31	[16,17,19,21,24,25,27,30,32,37,39,41,45,51,52,62,63,64,65,67,69,71,72,74,75,82,83,84,86,88,93]
Assisting Hand Assessment (AHA)	25	[13,14,21,26,32,38,47,48,49,51,53,54,56,61,63,93,94,95,96,97,98,99,100,101,102]
Box and Block Test (BBT)	15	[19,20,25,26,27,28,51,63,80,89,98,100,101,103,104]
Jebson-Taylor Hand Function Test (JTHFT)	12	[27,32,46,47,51,61,95,100,101,102,103,105]
ABILHAND-kids	12	[13,14,19,21,28,36,46,51,53,96,102,103]
Quality of Upper Extremity Skills Test (QUEST)	10	[32,39,40,43,61,62,67,72,94,106]
The Modified Ashworth Scale (MAS)	8	[39,56,73,76,98,107,108,109]
The Melbourne Assessment of Unilateral Upper Limb Function (MUUL)	7	[13,14,48,49,96,101,108]
Minute Walk Test (1, 2, 6, or 10)	6	[19,55,80,93,110,111]
Peabody Developmental Motor Scales (PDMS)	5	[15,43,74,106,112]
Bruininks-Oseretsky Test of Motor Proficiency (BOT)	5	[29,80,83,95,113]
Performance Quality Rating Scale (PQRS)	5	[30,37,42,89,114]

**Table 3:** GDT framework themes: frequency and source articles.

Theme	Count	Percentage	Citations
Theme 1: Collaborative Goal Setting	123	79%	[2,13,14,15,16,17,18,19,20,21,22,23,24,25,26,27,28,29,30,31,32,33,34,35,36,37,38,39,40,41,42,43,44,45,46,47,48,49,50,51,52,53,54,55,56,57,58,59,60,61,62,63,64,65,66,67,68,69,70,71,72,73,74,75,76,77,78,79,81,82,83,84,86,88,89,90,91,99,100,101,102,105,106,108,112,114,115,117,118,119,120,121,122,123,124,125,126,127,128,129,130,131,132,133,134,135,136,137,138,139,140,141,142,143,144,145,146,147,148,149,150,151]
Theme 2: Family-Centered Practice	90	58%	[2,13,14,15,16,17,18,19,23,24,26,27,32,33,34,36,37,40,41,42,44,47,48,49,52,53,54,55,56,57,59,61,63,65,66,67,68,69,70,71,72,74,75,76,77,79,81,88,89,90,95,97,99,100,101,105,106,113,115,116,117,119,120,121,122,123,124,125,126,128,129,131,134,135,136,137,139,142,145,147,148,149,151,152,153,154,155,156,157,158]
Theme 3: Specific Training Techniques	104	67%	[2,13,14,15,16,18,19,20,21,22,24,26,27,28,29,31,32,33,34,35,36,40,41,42,47,48,49,50,52,53,54,55,57,59,60,61,63,65,68,69,75,76,77,78,79,80,82,85,86,87,88,90,91,92,93,94,95,97,98,99,100,101,102,103,104,105,106,108,109,110,111,112,114,116,118,119,120,125,127,128,129,130,131,132,134,135,136,137,139,140,143,145,146,147,148,151,152,155,156,157,159,160,161,162]
Theme 4: Therapy Dose	118	76%	[2,13,14,16,17,18,19,20,21,24,25,26,28,29,31,32,33,34,35,36,38,40,41,46,47,48,49,50,51,52,53,54,55,56,57,58,60,61,63,67,68,69,70,71,72,73,74,75,76,77,78,80,81,82,83,85,86,87,88,89,90,91,92,93,94,95,96,97,99,100,101,102,103,104,105,106,107,108,109,110,111,112,113,114,115,116,117,118,119,121,123,124,126,127,128,129,130,131,132,133,135,137,138,139,141,144,146,147,148,151,154,156,157,158,160,162,163,164]
Intervention Studies	88		[13,14,16,17,18,19,20,21,24,25,26,27,28,29,31,32,33,34,35,36,38,40,41,46,47,48,49,50,51,52,53,54,55,56,57,58,60,61,63,67,68,69,70,71,72,73,74,75,76,77,78,80,81,82,83,85,86,87,88,89,90,91,92,93,94,95,96,97,99,100,101,102,103,104,105,106,107,108,109,110,111,112,113,114,116,147,160,162]
Qualitative Studies	31		[2,115,117,118,119,121,123,124,126,127,128,129,130,131,132,133,135,137,138,139,141,144,146,148,151,154,156,157,158,163,164]
Theme 5: Social Engagement	28	19%	[13,30,31,41,44,48,52,63,65,69,71,75,76,83,86,88,93,120,127,128,132,137,138,147,151,152,158,162]
Theme 6: Multidisciplinary Approaches	46	30%	[14,19,21,25,31,39,40,41,43,46,48,49,50,53,62,63,64,65,70,71,74,75,83,85,92,93,94,99,100,101,108,120,123,128,129,130,131,136,137,138,145,148,152,153,156,161]
Home-Based Therapy	31		[14,21,25,39,40,43,50,53,62,65,70,71,74,75,83,94,99,100,101,120,123,128,129,130,131,137,138,145,148,152,153]
School-Based Therapy	24		[14,25,31,39,41,48,50,63,64,65,71,74,75,83,93,94,120,129,136,137,138,153,156,161]
Theme 7: Outcome Monitoring	24	15%	[2,14,37,39,40,41,43,63,66,67,69,75,77,78,99,108,113,114,118,131,136,147,152,160]

**Table 4:** Goal-directed training process steps, specific guidelines, and associated themes.

Steps	Specific Guidelines	Associated Themes
1. Goal Setting: Identify individualized goals	1a: The collaborative approach begins with multiple stakeholders, including the client, parent, teacher, and therapy team.	Collaborative goal setting, Family-centered, Multidisciplinary approaches
	1b: The goals should have a functional focus and should target meaningful activities.	
	1c: The goals should be defined and measurable.	
2. Goal Analysis: Identify goal limiting factors	2a: It is important to determine what skills are needed, which skills are difficult, and which skills are strengths. These will be assessed through baseline measurements.	Family-centered, Social engagement, Multidisciplinary Approach, Outcome monitoring (2a)
	2b: Task analysis will need to be performed to determine any barriers and facilitators. The provider will need to consider multiple domains which can include motor, cognition, social, communication, and the environment.	
3. Determine Strategies and Practice	3a: The provider will then need to determine the approach. The approach can be remedial, accommodative, include modifications, mixed, evidence-based, or family preference depending on their needs.	Family-centered, Specific training techniques, Therapy dose (3d), Social engagement (3c), Multidisciplinary approach
	3b: When determining the activities and tasks, the provider will need to provide the family and child with motivation, seeking out the best way to engage them, and finding the just right challenge.	
	3c: The provider will then need to determine the environmental supports including the different domains of social, physical, modifications, products, and technology.	
	3d: The provider will determine the dose, which is the amount of time to practice the skill or goal together.	
	3e: The provider will need to educate the family and client on the intervention strategies through practicing the skill or goal together.	
4. Targeted Practice of the Skill/Goal		Family-centered (especially at home), Specific training techniques, Therapy dose, Social engagement (depending on if their goal is social or performed in social contexts), Multidisciplinary approach
5. Provide Feedback and Progressive Tailoring	Continue step 4 throughout this process while in collaboration with the therapy team, the family, and the client.	Family-centered, Specific training techniques, Therapy dose, Social engagement (depending on if their goal is social or performed in social contexts), Multidisciplinary approach, Outcome monitoring
6. Re-evaluate, Progress Tracking	Continue to collaborate with the therapy team, the family, and the client. At this time, it is important to re-evaluate Step 2 ‘Goal Analysis’ as needed, depending on the client’s progress with the skill or goal.	Family-centered, Specific training techniques, Therapy dose, Social engagement (because you go back to step 2), Multidisciplinary approach, Outcome monitoring
7. Practice Goal Across Contexts	Continue practice until mastery of the goal is achieved within different contexts of their daily life.	Family-centered, Specific training techniques, Therapy dose, Social engagement, Multidisciplinary approach
8. Evaluate Need for New Goals		Collaborative goal setting, Family-centered, Multidisciplinary approach, Outcome monitoring

## Data Availability

Not applicable.

## Supplementary Materials

The following supporting information can be downloaded at: https://www.mdpi.com/article/10.3390/children12081039/s1, Table S1: Outcome Measures, ICF Domains, and Subdomains.

## References

[1] Rosenbaum P., Paneth N., Leviton A., Goldstein M., Bax M., Damiano D., Dan B., Jacobsson B. (2007). A report: The definition and classification of cerebral palsy April 2006. Dev. Med. Child Neurol. Suppl..

[2] Jackman M., Sakzewski L., Morgan C., Boyd R.N., Brennan S.E., Langdon K., Toovey R.A.M., Greaves S., Thorley M., Novak I. (2022). Interventions to improve physical function for children and young people with cerebral palsy: International clinical practice guideline. Dev. Med. Child Neurol..

[3] Novak I., Paton M.C., Finch-Edmondson M., Badawi N., Fahey M., Velde A., Hines A., Dark L., Khamis A., Mc Namara M. (2021). Commentary and clinical implications of “state of the evidence traffic lights 2019: Systematic review of interventions for preventing and treating children with cerebral palsy”. J. Exp. Neurol..

[4] Jackman M., Thorley M., Toovey R., Burgess A., Blatch-Williams R., Sakzewski L., Boyd R.N., Novak I. (2025). Implementing Clinical Practice Guidelines for Improving Function in Cerebral Palsy: Development of a Fidelity Tool. Pediatr. Phys. Ther..

[5] Ogilvie L., Garbellini S., Sakzewski L., Davidson S.-A., Elliottt C. (2025). Key elements of Goal-Directed Training for children with cerebral palsy: A qualitative content analysis. Br. J. Occup. Ther..

[6] Hoffmann T.C., Glasziou P.P., Boutron I., Milne R., Perera R., Moher D., Altman D.G., Barbour V., Macdonald H., Johnston M. (2014). Better reporting of interventions: Template for intervention description and replication (TIDieR) checklist and guide. BMJ.

[7] Arksey H., O’Malley L. (2005). Scoping studies: Towards a methodological framework. Int. J. Soc. Res. Methodol..

[8] Shierk A., Roberts H., O’Brien A., Thias B.B. (2023). Scoping Review of Goal Directed Therapy for Youth with Cerebral Palsy. https://osf.io/5t3bq.

[9] Novak I., McIntyre S., Morgan C., Campbell L., Dark L., Morton N., Stumbles E., Wilson S.A., Goldsmith S. (2013). A systematic review of interventions for children with cerebral palsy: State of the evidence. Dev. Med. Child Neurol..

[10] Novak I., Morgan C., Fahey M., Finch-Edmondson M., Galea C., Hines A., Langdon K., Namara M.M., Paton M.C., Popat H. (2020). State of the Evidence Traffic Lights 2019: Systematic Review of Interventions for Preventing and Treating Children with Cerebral Palsy. Curr. Neurol. Neurosci. Rep..

[11] Lambert V.A., Lambert C.E. (2013). Qualitative Descriptive Research: An Acceptable Design. Pac. Rim Int. J. Nurs. Res..

[12] World Health Organization (2001). International Classification of Functioning, Disability and Health: ICF.

[13] Aarts P.B., Jongerius P.H., Geerdink Y.A., Van Limbeek J., Geurts A.C. (2010). Effectiveness of modified constraint-induced movement therapy in children with unilateral spastic cerebral palsy: A randomized controlled trial. Neurorehabilit. Neural. Repair..

[14] Aarts P.B., van Hartingsveldt M., Anderson P.G., van den Tillaar I., van der Burg J., Geurts A.C. (2012). The pirate group intervention protocol: Description and a case report of a modified constraint-induced movement therapy combined with bimanual training for young children with unilateral spastic cerebral palsy. Occup. Ther. Int..

[15] Morgan C., Novak I., Dale R., Badawi N. (2015). Optimising motor learning for infants at high risk of cerebral palsy: A pilot study. BMC Pediatr..

[16] Armstrong E., Kentish M., Boyd R., Horan S., Carty C. (2020). A randomized controlled trial of functional electrical stimulation powered cycling, goaldirected training and adapted cycling in children with cerebral palsy. Dev. Med. Child Neurol..

[17] Armstrong E.L., Boyd R.N., Horan S.A., Kentish M.J., Ware R.S., Carty C.P. (2022). Maintenance of Functional Gains Following a Goal-Directed and FES-Assisted Cycling Program for Children With Cerebral Palsy. Pediatr. Phys. Ther..

[18] Arnevik Austrheim K., Skagen C., Rieber J., Melfald Tveten K. (2024). Practice, play, repeat–individualized outcomes after the “intensity matters!”-program for children with disabilities–a descriptive multicase study. Disabil. Rehabil..

[19] Bleyenheuft Y., Ebner-Karestinos D., Surana B., Paradis J., Sidiropoulos A., Renders A., Friel K.M., Brandao M., Rameckers E., Gordon A.M. (2017). Intensive upper-and lower-extremity training for children with bilateral cerebral palsy: A quasi-randomized trial. Dev. Med. Child Neurol..

[20] Bono G.L.P., Achermann P., Rückriem B., Lieber J., van Hedel H.J.A. (2022). Goal-directed personalized upper limb intensive therapy (PULIT) for children with hemiparesis: A retrospective analysis. Am. J. Occup. Ther..

[21] Brandão M.B., Ferre C., Kuo H.C., Rameckers E.A.A., Bleyenheuft Y., Hung Y.C., Friel K., Gordon A.M. (2014). Comparison of structured skill and unstructured practice during intensive bimanual training in children with unilateral spastic cerebral palsy. Neurorehabilit. Neural. Repair..

[22] Branjerdporn N., Ziviani J., Sakzewski L. (2018). Goal-directed occupational therapy for children with unilateral cerebral palsy: Categorising and quantifying session content. Br. J. Occup. Ther..

[23] Cusick A., McIntyre S., Novak I., Lannin N., Lowe K. (2006). A comparison of goal attainment scaling and the Canadian Occupational Performance Measure for paediatric rehabilitation research. Pediatr. Rehabil..

[24] de Brito Brandao M., Gordon A.M., Mancini M.C. (2012). Functional impact of constraint therapy and bimanual training in children with cerebral palsy: A randomized controlled trial. AJOT Am. J. Occup. Ther..

[25] Feitosa A.M., Mancini M.C., Silvério A.P.M., Gordon A.M., Brandão M.B. (2021). “help me to improve my own priorities!”: A feasibility study of an individualized intensive goal training for adolescents with cerebral palsy. Phys. Occup. Ther. Pediatr..

[26] Ferre C.L., Brandão M., Surana B., Dew A.P., Moreau N.G., Gordon A.M. (2017). Caregiver-directed home-based intensive bimanual training in young children with unilateral spastic cerebral palsy: A randomized trial. Dev. Med. Child Neurol..

[27] Figueiredo P.R.P., Mancini M.C., Feitosa A.M., Teixeira C.M.M.F., Guerzoni V.P.D., Elvrum A.K.G., Ferre C.L., Gordon A.M., BrandÃo M.B. (2020). Hand–arm bimanual intensive therapy and daily functioning of children with bilateral cerebral palsy: A randomized controlled trial. Dev. Med. Child Neurol..

[28] Geerdink Y., Aarts P., van der Burg J., Steenbergen B., Geurts A. (2015). Intensive upper limb intervention with self-management training is feasible and promising for older children and adolescents with unilateral cerebral palsy. Res. Dev. Disabil..

[29] Ghorbani N., Rassafiani M., Izadi-Najafabadi S., Yazdani F., Akbarfahimi N., Havaei N., Gharebaghy S. (2017). Effectiveness of cognitive orientation to (daily) occupational performance (CO-OP) on children with cerebral palsy: A mixed design. Res. Dev. Disabil..

[30] Gimeno H., Polatajko H.J., Cornelius V., Lin J.P., Brown R.G. (2021). Rehabilitation in childhood-onset hyperkinetic movement disorders including dystonia: Treatment change in outcomes across the ICF and feasibility of outcomes for full trial evaluation. Eur. J. Paediatr. Neurol..

[31] Haddon M., West L., Elliott C., Walmsley C., Valentine J., Bear N., Pool D. (2023). Kindy Moves: The feasibility of an intensive interdisciplinary programme on goal and motor outcomes for preschool-aged children with neurodisabilities requiring daily equipment and physical assistance. BMJ Open.

[32] Hoare B., Imms C., Carey L. (2010). Intensive Upper Limb Therapy Following Botulinum-A in Young Children with Hemiplegic Cerebral Palsy: Results from a Randomised Controlled Trial. Dev. Med. Child Neurol..

[33] Imms C., Mathews S., Nicola Richmond K., Law M., Ullenhag A. (2016). Optimising leisure participation: A pilot intervention study for adolescents with physical impairments. Disabil. Rehabil..

[34] Jackman M., Novak I., Lannin N., Froude E., Miller L., Galea C. (2018). Effectiveness of cognitive orientation to daily occupational performance over and above functional hand splints for children with cerebral palsy or brain injury: A randomized controlled trial. BMC Pediatr..

[35] Jackman M., Novak I., Lannin N.A., Galea C., Froude E. (2018). The Cognitive Orientation to daily Occupational Performance (CO-OP) Approach: Best responders in children with cerebral palsy and brain injury. Res. Dev. Disabil..

[36] Janssen-Potten Y.J.M., Roks L., Roijen R., Vermeulen R.J., Adelante Study G., Rameckers E.E.A. (2023). Effectiveness of functional intensive therapy on mobility and self-care activities in children and adolescents with cerebral palsy–a prospective clinical study. Disabil. Rehabil..

[37] Lammi B.M., Law M. (2003). The effects of family-centred functional therapy on the occupational performance of children with cerebral palsy. Can. J. Occup. Ther..

[38] Lidman G., Nachemson A., Peny-Dahlstrand M., Himmelmann K. (2015). Botulinum toxin A injections and occupational therapy in children with unilateral spastic cerebral palsy: A randomized controlled trial. Dev. Med. Child Neurol..

[39] Lowe K., Novak I., Cusick A. (2006). Low-dose/high-concentration localized botulinum toxin A improves upper limb movement and function in children with hemiplegic cerebral palsy. Dev. Med. Child Neurol..

[40] Novak I., Cusick A., Lannin N. (2009). Occupational therapy home programs for cerebral palsy: Double-blind, randomized, controlled trial. Pediatrics.

[41] Ödman P.E., Öberg B.E. (2006). Effectiveness and expectations of intensive training: A comparison between child and youth rehabilitation and conductive education. Disabil. Rehabil..

[42] Öhrvall A.-M., Hofgren C., Lindquist B., Bergqvist L., Himmelmann K., Opheim A., Sjöwall D., Brock K., Peny-Dahlstrand M. (2024). Intervention with the CO-OP Approach leads to a transfer effect over time to untrained goals for children with cerebral palsy or spina bifida. Disabil. Rehabil..

[43] Olesch C.A., Greaves S., Imms C., Reid S.M., Graham H.K. (2010). Repeat botulinum toxin-A injections in the upper limb of children with hemiplegia: A randomized controlled trial. Dev. Med. Child Neurol..

[44] Palisano R.J., Chiarello L.A., King G.A., Novak I., Stoner T., Fiss A. (2012). Participation-based therapy for children with physical disabilities. Disabil. Rehabil..

[45] Pollock N., Sharma N., Christenson C., Law M., Gorter J.W., Darrah J. (2014). Change in parent-identified goals in young children with cerebral palsy receiving a context-focused intervention: Associations with child, goal and intervention factors. Phys. Occup. Ther. Pediatr..

[46] Roelofsma R., Rameckers E. (2017). The effect of a functional intensive intervention program on self-care in children with cerebral palsy: A case study. Int. J. Brain Disord. Treat.

[47] Sakzewski L., Provan K., Ziviani J., Boyd R.N. (2015). Comparison of dosage of intensive upper limb therapy for children with unilateral cerebral palsy: How big should the therapy pill be?. Res. Dev. Disabil..

[48] Sakzewski L., Ziviani J., Abbott D.F., Macdonell R.A., Jackson G.D., Boyd R.N. (2011). Participation Outcomes in a Randomized Trial of 2 Models of Upper-Limb Rehabilitation for Children With Congenital Hemiplegia. Arch. Phys. Med. Rehabil..

[49] Sakzewski L., Ziviani J., Boyd R.N. (2011). Best responders after intensive upper-limb training for children with unilateral cerebral palsy. Arch. Phys. Med. Rehabil..

[50] Sanli B.B., Janssen-Potten Y.J.M., Meeuwsen I., Coenen M., Caponi L., Roijen R., Teeuwen L., van den Berge G., de Haan C., Steinbusch C. (2020). Effect on quality of life in children and adolescents with disabilities after a functional intensive therapy approach. Biomed. J. Sci. Tech. Res..

[51] Saussez G., Bailly R., Araneda R., Paradis J., Ebner-Karestinos D., Klöcker A., Sogbossi E.S., Riquelme I., Brochard S., Bleyenheuft Y. (2023). Efficacy of integrating a semi-immersive virtual device in the HABIT-ILE intervention for children with unilateral cerebral palsy: A non-inferiority randomized controlled trial. J. Neuroeng. Rehabil..

[52] Sel S.A., Günel M.K., Erdem S., Tunçdemir M. (2023). Effects of Telerehabilitation-Based Structured Home Program on Activity, Participation and Goal Achievement in Preschool Children with Cerebral Palsy: A Triple-Blinded Randomized Controlled Trial. Children.

[53] Speth L., Janssen-Potten Y., Rameckers E., Defesche A., Winkens B., Becher J., Smeets R., Vles H. (2015). Effects of botulinum toxin A and/or bimanual task-oriented therapy on upper extremity activities in unilateral Cerebral Palsy: A clinical trial. BMC Neurol..

[54] Steinbusch C.V.M., Defesche A., van der Leij B., Rameckers E.A.A., Knijnenburg A.C.S., Vermeulen J.R.J., Janssen-Potten Y.J.M. (2023). The Effect of Bimanual Intensive Functional Training on Somatosensory Hand Function in Children with Unilateral Spastic Cerebral Palsy: An Observational Study. J. Clin. Med..

[55] Thomas R.E., Johnston L.M., Sakzewski L., Kentish M.J., Boyd R.N. (2016). Evaluation of group versus individual physiotherapy following lower limb intra-muscular Botulinum Toxin-Type A injections for ambulant children with cerebral palsy: A single-blind randomized comparison trial. Res. Dev. Disabil..

[56] Wallen M., Ziviani J., Naylor O., Evans R., Novak I., Herbert R.D. (2011). Modified constraint-induced therapy for children with hemiplegic cerebral palsy: A randomized trial. Dev. Med. Child Neurol..

[57] Balci N.C., Günel M.K., Erden Z., Tekindal M.A. (2021). Effect of Goal Directed Phys-iotherapy vs a Goal Directed Home Program in At-Risk Infants: A Randomized Clinical Trial. Adv. Neurol. Neurosci..

[58] Elliott C.M., Reid S.L., Alderson J.A., Elliott B.C. (2011). Lycra arm splints in conjunction with goal-directed training can improve movement in children with cerebral palsy. NeuroRehabilitation.

[59] Franki I., Van den Broeck C., De Cat J., Tijhuis W., Molenaers G., Vanderstraeten G., Desloovere K. (2014). A randomized, single-blind cross-over design evaluating the effectiveness of an individually defined, targeted physical therapy approach in treatment of children with cerebral palsy. Clin. Rehabil..

[60] Gibson N., Chappell A., Blackmore A.M., Morris S., Williams G., Bear N., Allison G. (2018). The effect of a running intervention on running ability and participation in children with cerebral palsy: A randomized controlled trial. Disabil. Rehabil..

[61] Gordon A.M., Hung Y.C., Brandao M., Ferre C.L., Kuo H.C., Friel K., Petra E., Chinnan A., Charles J.R. (2011). Bimanual training and constraint-induced movement therapy in children with hemiplegic cerebral palsy: A randomized trial. Neurorehabilit. Neural. Repair..

[62] Kawamura A., Campbell K., Lam-Damji S. (2007). A Randomized Controlled Trial Comparing Botulinum Toxin A Dosage in the Upper Extremity of Children with Spasticity. Dev. Med. Child Neurol..

[63] Knight S., Fetters L. (2010). Intensive Motor Skills Training Program Combining Group and Individual Sessions for Children With Cerebral Palsy. Pediatr. Phys. Ther..

[64] Lööwing K., Hamer E.G., Bexelius A., Carlberg E.B. (2011). Exploring the relationship of family goals and scores on standardized measures in children with cerebral palsy, using the ICF-CY. Dev. Neurorehabilit..

[65] Löwing K., Bexelius A., Brogren Carlberg E. (2009). Activity focused and goal directed therapy for children with cerebral palsy—Do goals make a difference?. Disabil. Rehabil..

[66] Löwing K., Bexelius A., Carlberg E.B. (2010). Goal-directed functional therapy: A longitudinal study on gross motor function in children with cerebral palsy. Disabil. Rehabil..

[67] Novak I., Cusick A., Lowe K. (2007). A pilot study on the impact of occupational therapy home programming for young children with cerebral palsy. Am. J. Occup. Ther..

[68] Palee S., Ploypetch T., Pajareya K., Timdang S. (2022). Goal-Directed Therapy to Improve Gross Motor Function and the Quality of Life of Children with Cerebral Palsy: A Randomized Controlled Trial. Siriraj Med. J..

[69] Salavati M., Rameckers E., Waninge A., Krijnen W., van der Schans C., Steenbergen B. (2018). Evaluating the outcome of an individual functional therapy program focused on children with cerebral palsy and cerebral visual impairment: A multiple case study. Eur. J. Physiother..

[70] Seyhan K., GÜNel M.K., AkyÜZ E.Ü. (2020). Family-centred, goal-directed multidisciplinary approach for lower extremity botulinum toxin with physical therapy and rehabilitation in cerebral palsy. Türk Fiz. Ve Rehabil. Derg..

[71] Sørensen K., Vestrheim I.E., Lerdal B., Skranes J. (2020). Functional Skills among Preschool Children with Cerebral Palsy–Assessment before and after Early Intervention. Dev. Neurorehabilit..

[72] Sorsdahl A.B., Moe-Nilssen R., Kaale H.K., Rieber J., Strand L.I. (2010). Change in basic motor abilities, quality of movement and everyday activities following intensive, goal-directed, activity-focused physiotherapy in a group setting for children with cerebral palsy. BMC Pediatr..

[73] Türker D., Korkem D., Özal C., Günel M.K., Karahan S. (2015). The effects of neurodevelopmental (Bobath) therapy based goal directed therapy on gross motor function and functional status of children with cerebral palsy. Int. J. Ther. Rehabil. Res..

[74] Wu C.-L., Liao S.-F., Liu C.-H., Hsieh Y.-T., Lin Y.-R. (2020). A pilot study of two different constraint-induced movement therapy interventions in children with hemiplegic cerebral palsy after botulinum toxin injection during preschool education. Front. Pediatr..

[75] Ahl L.E., Johansson E., Granat T., Carlberg E.B. (2005). Functional therapy for children with cerebral palsy: An ecological approach. Dev. Med. Child Neurol..

[76] Akbari A., Javadzadeh M., Shahraki G., Jahanshahi J.P. (2009). The effects of functional therapy on motor development in children with cerebral palsy. Iran. J. Child Neurol..

[77] Al Imam M.H., Jahan I., Das M.C., Bashar S.M.K., Khan A., Muhit M., Power R., Akbar D., Badawi N., Khandaker G. (2023). SUpporting People in extreme POverty with Rehabilitation and Therapy (SUPPORT CP): A trial among families of children with cerebral palsy in Bangladesh. Dev. Med. Child Neurol..

[78] Bower E., Michell D., Burnett M., Campbell M.J., McLellan D.L. (2001). Randomized controlled trial of physiotherapy in 56 children with cerebral palsy followed for 18 months. Dev. Med. Child Neurol..

[79] Brit Sorsdahl A., Moe-Nilssen R., Larsen E.M., Lundal S.H., Rieber J., Skarstein E., Kaale H.K., Strand L.I. (2020). Long-term change of gross motor function in children with cerebral palsy; an observational study of repeated periods of intensive physiotherapy in a group setting. Eur. J. Physiother..

[80] Crompton J., Imms C., McCoy A.T., Randall M., Eldridge B., Scoullar B., Galea M.P. (2007). Group-based task-related training for children with cerebral palsy: A pilot study. Phys. Occup. Ther. Pediatr..

[81] Heathcock J.C., Baranet K., Ferrante R., Hendershot S. (2015). Daily Intervention for Young Children with Cerebral Palsy in GMFCS Level V: A Case Series. Pediatr. Phys. Ther..

[82] Ketelaar M., Vermeer A., Hart H.t., van Petegem-van Beek E., Helders P.J.M. (2001). Effects of a functional therapy program on motor abilities of children with cerebral palsy. Phys. Ther..

[83] Ko E.J., Sung I.Y., Moon H.J., Yuk J.S., Kim H.-S., Lee N.H. (2020). Effect of group-task-oriented training on gross and fine motor function, and activities of daily living in children with spastic cerebral palsy. Phys. Occup. Ther. Pediatr..

[84] Law M.C., Darrah J., Pollock N., Wilson B., Russell D.J., Walter S.D., Rosenbaum P., Galuppi B. (2011). Focus on function: A cluster, randomized controlled trial comparing child-versus context-focused intervention for young children with cerebral palsy. Dev. Med. Child Neurol..

[85] Lee S.H., Shim J.S., Kim K., Moon J., Kim M. (2015). Gross motor function outcome after intensive rehabilitation in children with bilateral spastic cerebral palsy. Ann. Rehabil. Med..

[86] Myrhaug H.T., Odgaard-Jensen J., Jahnsen R. (2019). The long-term effects of conductive education courses in young children with cerebral palsy: A randomized controlled trial. Dev. Neurorehabilit..

[87] Sah A.K., Balaji G.K., Agrahara S. (2019). Effects of task-oriented activities based on neurodevelopmental therapy principles on trunk control, balance, and gross motor function in children with spastic diplegic cerebral palsy: A single-blinded randomized clinical trial. J. Pediatr. Neurosci..

[88] Saquetto M.B., de Santana Bispo A., da Silva Barreto C., Gonçalves K.A., Queiroz R.S., da Silva C.M., Gomes Neto M. (2018). Addition of an educational programme for primary caregivers to rehabilitation improves self-care and mobility in children with cerebral palsy: A randomized controlled trial. Clin. Rehabil..

[89] Sousa L.K., Brandão M.B., Curtin C.M., Magalhães L.C. (2020). A collaborative and cognitive-based intervention for young people with cerebral palsy. Can. J. Occup. Ther..

[90] Stark C., Duran I., Martakis K., Spiess K., Semler O., Schoenau E. (2020). Effect of long-term repeated interval rehabilitation on the gross motor function measure in children with cerebral palsy. Neuropediatrics.

[91] Bar-Haim S., Harries N., Nammourah I., Oraibi S., Malhees W., Loeppky J., Perkins N.J., Belokopytov M., Kaplanski J., Lahat E. (2010). Effectiveness of motor learning coaching in children with cerebral palsy: A randomized controlled trial. Clin. Rehabil..

[92] Wang L., Zhang N., Fang L., Cui Z., Niu H., Lv F., Hu D., Wu D. (2023). Effect of hip CPM on gross motor function and development of the hip joint: A single-center randomized controlled study on spastic cerebral palsy children with hip dysplasia. Front. Pediatr..

[93] Bleyenheuft Y., Bleyenheuft C., Arnould C., Brandao M.B., Gordon A.M. (2015). Hand and Arm Bimanual Intensive Therapy Including Lower Extremity (HABIT-ILE) in Children With Unilateral Spastic Cerebral Palsy: A Randomized Trial. Neurorehabilit. Neural. Repair..

[94] Gelkop N., Burshtein D.G., Lahav A., Brezner A., Al-Oraibi S., Ferre C.L., Gordon A.M. (2015). Efficacy of constraint-induced movement therapy and bimanual training in children with hemiplegic cerebral palsy in an educational setting. Phys. Occup. Ther. Pediatr..

[95] Gordon A.M., Schneider J.A., Chinnan A., Charles J.R. (2007). Efficacy of a hand–arm bimanual intensive therapy (HABIT) in children with hemiplegic cerebral palsy: A randomized control trial. Dev. Med. Child Neurol..

[96] Kirton A., Andersen J., Herrero M., Nettel-Aguirre A., Carsolio L., Damji O., Keess J., Mineyko A., Hodge J., Hill M.D. (2016). Brain stimulation and constraint for perinatal stroke hemiparesis: The PLASTIC CHAMPS Trial. Neurology.

[97] Klevberg G.L., Zucknick M., Jahnsen R., Eliasson A.C. (2023). Development of Hand Use with and Without Intensive Training Among Children with Unilateral Cerebral Palsy in Scandinavia. Dev. Neurorehabilit..

[98] Lieber J., Dittli J., Lambercy O., Gassert R., Meyer-Heim A., Hubertus J.A.v.H. (2022). Clinical utility of a pediatric hand exoskeleton: Identifying users, practicability, and acceptance, and recommendations for design improvement. J. Neuroeng. Rehabil..

[99] Palomo-Carrión R., Lirio-Romero C., Ferri-Morales A., Jovellar-Isiegas P., Cortés-Vega M.-D., Romay-Barrero H. (2021). Combined intensive therapies at home in spastic unilateral cerebral palsy with high bimanual functional performance. What do they offer? A comparative randomised clinical trial. Ther. Adv. Chronic Dis..

[100] Roldán-Pérez P., Abuín-Porras V., Buesa-Estéllez A., Ortiz-Lucas M. (2022). Functional Splinting efficacy in a Specific Task Home Program for Children with Cerebral Palsy. A Randomized Controlled Trial. Dev. Neurorehabilit..

[101] Sakzewski L., Miller L., Ziviani J., Abbott D.F., Rose S., Macdonell R.A.L., Boyd R.N. (2015). Randomized comparison trial of density and context of upper limb intensive group versus individualized occupational therapy for children with unilateral cerebral palsy. Dev. Med. Child Neurol..

[102] Smorenburg A.R.P., Gordon A.M., Kuo H.-C., Ferre C.L., Brandao M., Bleyenheuft Y., Carmel J.B., Friel K.M. (2017). Does corticospinal tract connectivity influence the response to intensive bimanual therapy in children with unilateral cerebral palsy?. Neurorehabilit. Neural. Repair..

[103] Green D., Wilson P.H. (2012). Use of virtual reality in rehabilitation of movement in children with hemiplegia—A multiple case study evaluation. Disabil. Rehabil..

[104] Song C.-S. (2014). Effects of task-oriented approach on affected arm function in children with spastic hemiplegia due to cerebral palsy. J. Phys. Ther. Sci..

[105] Sakzewski L., Ziviani J., Abbott D.F., Macdonell R.A.L., Jackson G.D., Boyd R.N. (2011). Randomized trial of constraint-induced movement therapy and bimanual training on activity outcomes for children with congenital hemiplegia. Dev. Med. Child Neurol..

[106] Kanitkar A., Szturm T., Parmar S., Gandhi D.B., Rempel G.R., Restall G., Sharma M., Narayan A., Pandian J., Naik N. (2017). The Effectiveness of a Computer Game-Based Rehabilitation Platform for Children With Cerebral Palsy: Protocol for a Randomized Clinical Trial. JMIR Res. Protoc..

[107] Glavić J., Rutović S., Cvitanović N.K., Burić P., Petrović A. (2016). Technology-enhanced upper limb physical rehabilitation in hemiplegic cerebral palsy. Int. J. Neurorehabilit..

[108] Rameckers E.A., Duysens J., Speth L.A., Vles H.J., Smits-Engelsman B.C. (2010). Effect of addition of botulinum toxin-A to standardized therapy for dynamic manual skills measured with kinematic aiming tasks in children with spastic hemiplegia. J. Rehabil. Med..

[109] Simon-Martinez C., Mailleux L., Jaspers E., Ortibus E., Desloovere K., Klingels K., Feys H. (2020). Effects of combining constraint-induced movement therapy and action-observation training on upper limb kinematics in children with unilateral cerebral palsy: A randomized controlled trial. Sci. Rep..

[110] Blundell S.W., Shepherd R.B., Dean C.M., Adams R.D., Cahill B.M. (2003). Functional strength training in cerebral palsy: A pilot study of a group circuit training class for children aged 4-8 years. Clin. Rehabil..

[111] Schasfoort F., Pangalila R., Sneekes E., Catsman C., Becher J., Horemans H., Stam H., Dallmeijer A., Bussmann H. (2018). Intramuscular botulinum toxin prior to comprehensive rehabilitation has no added value for improving motor impairments, gait kinematics and goal attainment in walking children with spastic cerebral palsy. J. Rehabil. Med..

[112] Melamalai S., Amalraj C., Alanazi A.O.K., Vadivel S., Senapati A. (2023). Splints And a Task-Oriented Approach Improve Upper Extremity Function in Children with Spastic Quadriplegic Cerebral Palsy. Int. J. Multidiscip. Res..

[113] Chen Y., Garcia-Vergara S., Howard A.M. (2015). Effect of a home-based virtual reality intervention for children with cerebral palsy using super pop VR evaluation metrics: A feasibility study. Rehabil. Res. Pract..

[114] Gimeno H., Polatajko H.J., Lin J.P., Cornelius V., Brown R.G. (2020). Cognitive Strategy Training in Childhood-Onset Movement Disorders: Replication Across Therapists. Front. Pediatr..

[115] Armstrong E.L., Boyd R.N., Carty C.P., Kentish M.J., Goodlich B.I., Horan S.A. (2022). A qualitative analysis of the experiences of children with cerebral palsy and their caregivers in a goal-directed cycling programme. Disabil. Rehabil..

[116] Miller L., Ziviani J., Ware R.S., Boyd R.N. (2016). Does context matter? Mastery motivation and therapy engagement of children with cerebral palsy. Phys. Occup. Ther. Pediatr..

[117] Vroland-Nordstrand K., Eliasson A.-C., Krumlinde-Sundholm L., Johansson U. (2018). Parents’ experiences of conducting a goal-directed intervention based on children’s self-identified goals, a qualitative study. Scand. J. Occup. Ther..

[118] Al Imam M.H., Jahan I., Muhit M., Das M.C., Power R., Khan A., Akbar D., Badawi N., Khandaker G. (2021). Supporting Ultra Poor People with Rehabilitation and Therapy among families of children with Cerebral Palsy in rural Bangladesh (SUPPORT CP): Protocol of a randomised controlled trial. PLoS ONE.

[119] Armstrong E.L., Boyd R.N., Kentish M.J., Carty C.P., Horan S.A. (2019). Effects of a training programme of functional electrical stimulation (FES) powered cycling, recreational cycling and goal-directed exercise training on children with cerebral palsy: A randomised controlled trial protocol. BMJ Open.

[120] Aydin R., Nur H. (2012). Family-centered approach in the management of children with cerebral palsy. Turk. J. Phys. Med. Rehabil./Turk. Fiz. Tip Ve Rehabil. Derg..

[121] Boyd R., Sakzewski L., Provan K., Abbott D.F., Badawy R., Gilmore R., Tournier J., Macdonell R.A.L., Jackson G.D., Ziviani J. (2010). INCITE: A randomised trial comparing constraint induced movement therapy and bimanual training in children with congenital hemiplegia. BMC Neurol..

[122] Hoare B., Greaves S. (2017). Unimanual versus bimanual therapy in children with unilateral cerebral palsy: Same, same, but different. J. Pediatr. Rehabil. Med..

[123] Imms C., Cowan R., Ertekin E., Klein G.-L., Galvin J. (2010). Eight weeks of occupational therapy home programme, compared to no programme, resulted in improved achievement of child and family-selected goals by children with cerebral palsy. Aust. Occup. Ther. J..

[124] Jackman M., Novak I., Lannin N. (2014). Effectiveness of functional hand splinting and the cognitive orientation to occupational performance (CO-OP) approach in children with cerebral palsy and brain injury: Two randomised controlled trial protocols. BMC Neurol..

[125] Klevberg G.L., Østensjø S., Elkjær S., Kjeken I., Jahnsen R.B. (2017). Hand function in young children with cerebral palsy: Current practice and parent-reported benefits. Phys. Occup. Ther. Pediatr..

[126] McLean B., Blakeman M., Carey L., Ward R., Novak I., Valentine J., Blair E., Taylor S., Bear N., Bynevelt M. (2018). Discovering the sense of touch: Protocol for a randomised controlled trial examining the efficacy of a somatosensory discrimination intervention for children with hemiplegic cerebral palsy. BMC Pediatr..

[127] Milton Y.M., Roe S.A., Newby K.V. (2020). Home programmes based on evidence of best practice for children with unilateral cerebral palsy: Occupational therapists’ perceptions. Br. J. Occup. Ther..

[128] Morgan C., Novak I., Dale R.C., Guzzetta A., Badawi N. (2014). GAME (Goals-Activity-Motor Enrichment): Protocol of a single blind randomised controlled trial of motor training, parent education and environmental enrichment for infants at high risk of cerebral palsy. BMC Neurol..

[129] Myrhaug H.T., Østensjø S. (2014). Motor training and physical activity among preschoolers with cerebral palsy: A survey of parents’ experiences. Phys. Occup. Ther. Pediatr..

[130] Novak I. (2014). Evidence-based diagnosis, health care, and rehabilitation for children with cerebral palsy. J. Child Neurol..

[131] Novak I., Berry J. (2014). Home program intervention effectiveness evidence. Phys. Occup. Ther. Pediatr..

[132] Novak I., Cusick A. (2006). Home programmes in paediatric occupational therapy for children with cerebral palsy: Where to start?. Aust. Occup. Ther. J..

[133] Oh T.-Y. (2019). Overview of Physical Therapy for Children with Cerebral Palsy. J. Korean Soc. Neurother..

[134] Öhrvall A.-M., Bergqvist L., Hofgren C., Peny-Dahlstrand M. (2020). “With CO-OP I’m the boss”–experiences of the cognitive orientation to daily occupational performance approach as reported by young adults with cerebral palsy or spina bifida. Disabil. Rehabil..

[135] Palomo-Carrión R., Pinero-Pinto E., Romay-Barrero H., Escobio-Prieto I., Lillo-Navarro C., Romero-Galisteo R.-P. (2022). Shall we start? Ready, set, go! Toward early intervention in infants with unilateral cerebral palsy. A randomized clinical trial protocol. Ther. Adv. Chronic Dis..

[136] Pritchard-Wiart L., Thompson-Hodgetts S., McKillop A.B. (2019). A review of goal setting theories relevant to goal setting in paediatric rehabilitation. Clin. Rehabil..

[137] Reedman S.E., Boyd R.N., Elliott C., Sakzewski L. (2017). ParticiPAte CP: A protocol of a randomised waitlist controlled trial of a motivational and behaviour change therapy intervention to increase physical activity through meaningful participation in children with cerebral palsy. BMJ Open.

[138] Sakzewski L., Bleyenheuft Y., Boyd R.N., Novak I., Elliott C., Reedman S., Morgan C., Pannek K., Fripp J., Golland P. (2019). Protocol for a multisite randomised trial of Hand–Arm Bimanual Intensive Training Including Lower Extremity training for children with bilateral cerebral palsy: HABIT-ILE Australia. BMJ Open.

[139] Sakzewski L., Pool D., Armstrong E., Reedman S.E., Boyd R.N., Elliott C., Novak I., Trost S., Ware R.S., Comans T. (2023). ACTIVE STRIDES-CP: Protocol for a randomised trial of intensive rehabilitation (combined intensive gait and cycling training) for children with moderate-to-severe bilateral cerebral palsy. BMJ Open.

[140] Sakzewski L., Ziviani J., Boyd R.N. (2014). Delivering evidence-based upper limb rehabilitation for children with cerebral palsy: Barriers and enablers identified by three pediatric teams. Phys. Occup. Ther. Pediatr..

[141] Sakzewski L., Ziviani J., Boyd R.N. (2016). Translating evidence to increase Quality and Dose of Upper Limb therapy for children with unilateral cerebral palsy: A pilot study. Phys. Occup. Ther. Pediatr..

[142] Saloojee G. (2022). The Akwenda cerebral palsy intervention programme. BMJ Open.

[143] Sansare A., Xanthopoulos M. (2022). Commentary on “Maintenance of Functional Gains Following a Goal-Directed and FES-Assisted Cycling Program for Children With Cerebral Palsy”. Pediatr. Phys. Ther..

[144] Saussez G., Brandão M.B., Gordon A.M., Bleyenheuft Y. (2017). Including a lower-extremity component during hand-arm bimanual intensive training does not attenuate improvements of the upper extremities: A retrospective study of randomized trials. Front. Neurol..

[145] Schnackers M., Beckers L., Janssen-Potten Y., Aarts P., Rameckers E., van der Burg J., de Groot I., Smeets R., Geurts S., COAD Focus Group (2018). Home-based bimanual training based on motor learning principles in children with unilateral cerebral palsy and their parents (the COAD-study): Rationale and protocols. BMC Pediatr..

[146] Størvold G.V., Jahnsen R.B. (2021). Current physical therapy practice in Norway for children with cerebral palsy. Pediatr. Phys. Ther..

[147] Tait K., Sigafoos J., Woodyatt G., O’Reilly M., Lancioni G. (2004). Evaluating parent use of functional communication training to replace and enhance prelinguistic behaviours in six children with developmental and physical disabilities. Disabil. Rehabil..

[148] Toovey R., Harvey A.R., McGinley J.L., Lee K.J., Shih S.T.F., Spittle A.J. (2018). Bike skills training for children with cerebral palsy: Protocol for a randomised controlled trial. BMJ Open.

[149] Toovey R., Spittle A.J., Nicolaou A., McGinley J.L., Harvey A.R. (2019). Training two-wheel bike skills in children with cerebral palsy: A practice survey of therapists in Australia. Phys. Occup. Ther. Pediatr..

[150] Ullenhag A., Jahnsen R., Klove N., Smedvig S., Hoberg A. (2024). How did youth with cerebral palsy perceive participation in everyday life after participating in a periodical intensive rehabilitation program based on adapted physical activity in groups? A qualitative interview study. Disabil. Rehabil..

[151] van Vulpen L.F., de Groot S., Rameckers E.A.A., Becher J.G., Dallmeijer A.J. (2017). Effectiveness of Functional Power Training on Walking Ability in Young Children With Cerebral Palsy: Study Protocol of a Double-Baseline Trial. Pediatr. Phys. Ther..

[152] Adiguzel H., Sarikabadayi Y.U., Elbasan B. (2023). Investigation of the effectiveness of family collaborative physiotherapy programs applied to high-risk infants. Physiother. Theory Pract..

[153] Andrews C., Kakooza-Mwesige A., Kamusiime S., Forssberg H., Eliasson A.C. (2023). A Goal-Directed Program for Wheelchair Use for Children and Young People with Cerebral Palsy in Uganda: An Explorative Intervention Study. J. Clin. Med..

[154] Araneda R., Sizonenko S.V., Newman C.J., Dinomais M., Le Gal G., Ebner-Karestinos D., Paradis J., Klöcker A., Saussez G., Demas J. (2020). Protocol of changes induced by early Hand-Arm Bimanual Intensive Therapy Including Lower Extremities (e-HABIT-ILE) in pre-school children with bilateral cerebral palsy: A multisite randomized controlled trial. BMC Neurol..

[155] Boyd R.N., Novak I., Morgan C., Bora S., Sakzewski L., Ware R.S., Comans T., Fahey M.C., Whittingham K., Trost S. (2023). Protocol: School readiness of children at high risk of cerebral palsy randomised to early neuroprotection and neurorehabilitation: Protocol for a follow-up study of participants from four randomised clinical trials. BMJ Open.

[156] Darrah J., Law M.C., Pollock N., Wilson B., Russell D.J., Walter S.D., Rosenbaum P., Galupp B. (2011). Context therapy: A new intervention approach for children with cerebral palsy. Dev. Med. Child Neurol..

[157] Johari S., Kahjoogh M.A., Nezhad Z.M., Hosseini S.A., Zamani Z.P., Shati M., Haghgoo H.A. (2020). Effects of transcranial direct current stimulation combined with cognitive orientation to daily occupational performance in children with cerebral palsy: A protocol for a randomised controlled trial. Int. J. Ther. Rehabil..

[158] Ödman P., Krevers B., Öberg B. (2007). Parents’ perceptions of the quality of two intensive training programmes for children with cerebral palsy. Dev. Med. Child Neurol..

[159] Bailes A.F., Greve K., Long J., Kurowski B., Vargus-Adams J., Aronow B., Mitelpunkt A. (2021). Describing the delivery of evidence-based physical therapy intervention to individuals with cerebral palsy. Pediatr. Phys. Ther. Off. Publ. Sect. Pediatr. Am. Phys. Ther. Assoc..

[160] Buitrago J.A., Bolaños A.M., Caicedo Bravo E. (2020). A motor learning therapeutic intervention for a child with cerebral palsy through a social assistive robot. Disabil. Rehabil. Assist. Technol..

[161] Ferro A.M., Quinn L. (2020). A structured goal-setting process to promote functional and measurable outcomes in school-based physical therapy: A knowledge translation study. Pediatr. Phys. Ther..

[162] Moreau N.G., Gannotti M.E. (2015). Addressing muscle performance impairments in cerebral palsy: Implications for upper extremity resistance training. J. Hand Ther..

[163] Fancourt D., Wee J., Lorencatto F. (2020). Identifying mechanisms of change in a magic-themed hand-arm bimanual intensive therapy programme for children with unilateral spastic cerebral palsy: A qualitative study using behaviour change theory. BMC Pediatr..

[164] Smidt K.B., Klevberg G.L., Oftedal B.F. (2020). Home Programme to Improve Hand Function for Children with Bilateral Cerebral Palsy: Beneficial but Challenging. Phys. Occup. Ther. Pediatr..

