# Supplementary material for: Defining Goal-Directed Training for Children with Cerebral Palsy: A Scoping Review and Framework for Implementation
Source: Children (Basel). 2025 Aug 8;12(8):1039. doi: 10.3390/children12081039 (PMC12384430; doi:10.3390/children12081039)
Supplement: Supplementary file 1 [file children-12-01039-s001.zip › children-3735105-supplementary.pdf]

Supplemental Material Table 1

Outcome Measures, ICF Domains, and Subdomains

Outcome Measure Abbreviation	Outcome Measure	ICF Domain	Subdomain
1MWT	1-Minute Walk Test	Body Structure/Function	Endurance
2MWT	2-Minute Walk Test	Body Structure/Function	Endurance
6MWT	6-Minute Walk Test	Body Structure/Function	Endurance
9HPT	9 Hole Peg Test	Activity	Dexterity
ABAS	Adaptive Behavior Assessment System<br>- Second Ed. (ABAS-II)	Activity and Participation	Psychological status, behavioral function, and social role participation
ABILHAND	ABILHAND – KIDS: Manual Ability Measure for Children with Upper Limb Impairments	Activity	Activities of daily living, dexterity
ABILOCO	ABILOCO – KIDS - Abilities in Locomotion Questionnaire	Activity	Locomotion
ACPC	Assessment of Preschool Children's Participation	Participation	Play, Skill development, physical recreation, social engagement
Activlim	Activlim – Activity Limitations Questionnaire	Activity	Activities of daily living
AHA	Assisting Hand Assessment	Activity	Gross and fine motor
AIMS	Alberta Infant Motor Scales	Activity	Gross motor
AMPS	Assessment of Motor and Process Skills	Activity	Motor and cognitive abilities

APS	Assistance to Participate Scale	Activity and Participation	Activities of daily living
AROM	active Range of Motion	Body Function	Mobility
ASEBA	The Achenbach System of Empirically Based Assessment	Body Function	Behavior
BBT	Box and Blocks test (BBT)	Body Structure/Function	Gross motor, dexterity
BFMDRS	Burke-Fahn-Marsden Dystonia Rating Scale	Body Structure/Function	Motor, activities of daily living
BoHA	Both Hands Assessment	Activity and Body Function	Gross and fine motor
BOT-2 or BOTMP	Bruininks-Oseretsky Test of Motor Proficiency<br>- Second edition (-2)	Activity	Gross motor and fine motor
BRIEF	Behavioral Rating Inventory of Executive Function	Body Function	Mental function
BSID-III	BAYLEY-III Scales of Infant and Toddler Development, and Third Addition	Activity	Motor, executive functioning, language
CAPE	Children's Assessment of Participation and Enjoyment	Participation	Participation in multiple domains
CAS	Caregiver Assistance Scale (PEDI)	Activity and Participation	Activities of daily living, mobility, and social/cognitive
CHEQ	Children's Hand-use Evaluation Questionnaire	Activity	Activities of daily living
CHORES	Children Helping Out: Responsibilities, Expectations and Supports	Activity and Participation	Instrumental activities of daily living
CHQ	Child Health Questionnaire	Participation	Health status
COPM	Canadian Occupational Performance Measure<br>- Performance (COPM-P)<br>- Satisfaction (COPM-S)	Activity and Participation	Occupational performance – self care, school/work, leisure

COSA	Child Occupational Self-Assessment	Activity	Activities of daily living and fine motor
CP-QOL	The Cerebral Palsy Quality of Life Questionnaire	Participation, Environment, Body function	Quality of life
DASS	Depression, Anxiety and Stress Scales<br>- short 21 item version (DASS-21)	Participation	Psychological status
DMQ	Dimensions of Mastery Questionnaire	Participation	Psychological status, motivation
EQ-5D	European Quality of Life – 5 Dimensions (Visual Analogue Scale)<br>- Levels of severity (3l, 5L)	Participation	Mobility, self-care, usual activities, pain/discomfort and anxiety/depression
EVGS	Edinburgh Visual Gait Score	Activity	Balance and coordination, gross motor
FAQ	Gillette Functional Assessment Questionnaire	Activity and Body function	Mobility, motor
FES	Family Environment Scale	Environment	Emotion
FIM or Wee-FIM	Functional Independence Measure for Children	Activity	Self-care, mobility, social cognition
FTSST	Five Times Sit-to-Stand Test	Activity	Functional mobility, balance
FMA	Fugl-Meyer	Activities, Body Structures and Function	Motor, sensory, balance, mobility, pain
GAS	Goal Attainment Scale	Activity	Goal achievement
GMFM	Gross Motor Function Measure<br>- 66<br>- 88	Activity	Gross motor
GMPM	Gross Motor Performance Measure	Activity	Gross motor

GMs	Prechtl's General Movements Assessment	Body structure	Neuro development
GMFCS	Gross motor function classification system	Activity and Participation	Functional participation
	Handwriting Speed Test	Activity, Body Function	Fine motor, executive functioning, visual perception
HHS	Harris Hip Score	Body function	Hip function and disability
HAT	Hypertonia Assessment Tool	Body structure and function	Tone
HiMAT	High Level Mobility Assessment Tool	Body function, activity	Functional mobility, vestibular
HINE	Hammersmith Infant Neurological Examination	Body structure and function	Motor patterns, reflexes, spontaneous movements, attention and behavior
HNNE	Hammersmith Neonatal Neurological Examination	Body Structure/ Function	Motor patterns, reflexes, spontaneous movements, attention and behavior
HOME – infant/toddler version	Home Observation Measurement of the Environment	Environment	Home environment
IPCA	Inventory of Potential Communicative Acts	Activity	Communication
Jebsen-Taylor	Jebsen Taylor Test of Hand Function	Activity	Fine motor
Kidscreen	Kidscreen - Health-Related Quality of Life Questionnaire for Children and Adolescents	Participation	Quality of life
Life-H	Assessment of Life Habits	Participation	Activities of daily living and social participation
MAS	Modified Ashworth Scale	Body Structure/Function	Tone

MA2	Melborne Assessment of Unilateral Upper Limb Function	Activity	Upper limb motor
MFPT	Manual Form Perception Test	Body Structure/Function	Stereognosis
MMT	Manual Muscle Testing	Body Structure/Function	Strength
MPOC	Measure of Processes of Care	Other	Measure of family-centered practice in providers
MPST	Muscle Power Sprint Test	Activity	Mobility
MST	10x5 Meter Sprint Test	Body Structure/Function	Endurance, mobility
MTS	Modified Tardieu Scale	Body structure/Function	Tone
MUUL	Melbourne Unilateral Upper Limb Function	Activity	Fine motor
NK Dexterity Board	NK Dexterity Board	Activity	Dexterity
OSAS	Observational Skills Assessment Score (capacity hand)	Body Structure/Function, Activity	Amount and quality of hand use
PAS	Postural Assessment Scale	Body Structure/Function	Posture, balance
PBS	Pediatric Balance Scale	Body Structure/Function	Posture, balance
PDMS	Peabody Developmental Motor Scales	Activity	Gross motor and fine motor
PEDI-CAT	Pediatric Evaluation of Disability Inventory – Computerized Assessment Tool	Activity	Daily activities, mobility, social/cognitive, and responsibility
PEDI-QL	Pediatric Quality of Life Inventory	Participation	Quality of life
PEM	Participation and Environment Measure<br>- Children and Youth (PEM-CY)	Participation, Environment, Activity	Multidomain
PMAL	Pediatric Motor Activity Log	Body Structure/Function	Activities of daily living, and motor
PQRS	Performance Quality Rating Scale<br>- Individualized (PDRS-i)	Activity	Motor

PROM	Passive Range of Motion	Body Function	Range of motion
PSFS	Patient Specific Functional Scale	Activity, Participation	Activities of daily living
PVQ	Pediatric Volitional Questionnaire	Activity, Participation	Multidomain
QUEST	Quality of Upper Extremity Skills Test	Activity	Motor
REEL-2	Receptive Expressive Emergent Language Scale	Activity	Receptive and expressive language
SCALE	Selective Control Assessment of Lower Extremity	Body Function	Isolated joint movement
SCUES	Selective Control of the Upper Extremity Scale	Body Function	Upper limb selective voluntary motor control
SFA	School Function Assessment	Activity and Participation	Multidomain
SMC	Selective Motor Control Scale	Body Function	Isolated joint movement
STS	Sit-To-Stand	Activity	Functional mobility, balance
SRT	10-meter Shuttle Run Test	Activity	Mobility, fitness
TAPQOL	TNO-AZL preschool children quality of life questionnaire	Body structures/functions and Activity	Quality of life
TARC Assessment System	Topeka Association for Retarded Citizens Assessment System	Body Structure/Function, Activity, Participation	Education, behavior, motor, social engagement, communication
TIS	Trunk Impairment Scale	Body structure/function and activity	Sitting balance, trunk coordination
TUG	Timed Up and Go Test	Activity	Balance and coordination
UL-3DMA	Upper Limb – Three-Dimensional Motion Analysis	Body Structure/Function	Gross and fine motor during specific tasks
VOAA	Video Observations Aarts and Aarts (VOAA)	Activity	Upper limb gross motor and fine

			motor (frequency, duration, and quality)
